# 

**Published:** 1878-06-20

**Authors:** 


ORIGINAL AND SELECTED ARTICLES.

Cystic Tumor of the Neck Treated with Ergot; by B. M. Walker, M D., of Va.........161
Obstinate Hemorrhage from Extraction of a Tooth; by S. H. Anderson, M.D., of Mo..163
Ustilago Maidis; by I. J. M. Goss, M. D., of Marietta, Ga............................................164
Review on the Treatment of Fracture of the Femur; by Edward Brock, M.D...............164
Placenta Prævia—Its Treatment; by Chas. F. A. Nichell, M.D., St. Helena, Cal.............169
Successful Treatment of a Case of Aneurism by the Use of the Esmarch Bandage........171
Society Reports—Atlanta Medico Chirurgical Association............................................172
Maternal Impressions.......................................174

ABSTRACTS AND GLEANINGS.

Treatment of Piles, 175; The Strong Elastic Bandage, 176; How to Deprive Iodine of its Stain, 176; Chlorate of Potash in Diarrhœa, 177; Esmarch on Cancer, 177; Koumiss, 178; Pulsatilla in Dysmenorrhœa, 178; Prophylaxis of Scarlatina, 179; Action of Ergot on the Uterus, 179; Alum in Aural Discharges, 179; A Strange Case, 180; Carbuncle, 180; Treatment of Ulcers, 180; Beer and Suicide, 181; Nitrite of Amyl in Asphyxia, 181; New Method of Dressing Stumps, 181; Exothalmia Treated by Galvanization, 182; Syphilis Transmitted by Vaccination, 182; Ovariotomy, 182; Treatment of Favus, 182; Muriate of Calcium in Tuberculosis, 182; Operative Treatment of Internal Piles, 183; Churchill's Tincture of Iodine, 183; Tonsilitis, 183; On the Frequent Connection between Eczema and Diabetes Mellitus, 184; Pregnancy and Suckling, 184; Treatment of Shingles by Topical Applications of Perchloride of Iron, 184; Carbolate of Soda in the Treatment of Nervous Affections of the Respiratory Passages, 184; Oil of Stavesacre in Scabies, 184; Fracture of the Femur, 185; Sarracenia Purpurea in Gout, 185; To Blister the Skin Extemporaneously, 185; Warm Baths in Traumatic Tetanus, 185.

PRACTICAL NOTES AND FORMULÆ.

Treatment of Alcoholism, 186; Early Rupture of Membranes, 186; Cod-Liver Oil with Hypophosphites and Whisky, 187; Boracic Acid Ointment, 187; Treatment of Mettorrhagia by Hypodermic Injections of Ergotine, 187; Neutralizing Cordial, 187; For Rigors, 187; Good Injection for Gonorrhœa, 187. A Durable Cement, 188; Rheumatic Tonic, 188; Hope's Dysenteric Mixture, 188; A Good Formula for the Summer Diarrhœas of Children, 188; Improved Chalk Mixture, 188; Carron Oil in Anal Fissure, 188; Chloral Dangerous in Delirium Tremens, 188; Dr. Atlee's Nipple Wash, 188.

SCIENTIFIC ITEMS.

Putrefaction, 189; Artificial Larynx, 189; An Army of Ants, 189; New Freezing Process, 189.

EDITORIAL AND MISCELLANEOUS.

Be Considerate, 190; Be Reasonable, 190; The No Stamp and Sample Copy Man, 190; The Record at the North, 190; Dr. Churchill Dead, 190; Be Just, 191; Hydrobromic Acid—Correction, 191; Nominating Committees, 191; Book Notices, 191; To Physicians and Druggists, 192; Notices of New Advertisements, 192.

